# Cultural concepts of distress among Albanian young adults living in Switzerland - a qualitative study

**DOI:** 10.3389/fpsyg.2024.1321452

**Published:** 2024-05-06

**Authors:** Imer Pnishi, Mirëlinda Shala, Naser Morina, Andreas Maercker, Eva Heim

**Affiliations:** ^1^Department of Psychology, Division of Psychopathology and Clinical Intervention, University of Zurich, Zürich, Switzerland; ^2^Department of Consultation-Liaison Psychiatry and Psychosomatic Medicine, University Hospital Zurich, University of Zurich, Zurich, Switzerland; ^3^Institute of Psychology, Division of Psychopathology and Clinical Intervention, University of Zurich, Zürich, Switzerland

**Keywords:** Albanian migrants, cultural concepts of distress, cultural identity, qualitative research, parentification, second-generation migrants, psychological distress, young adults

## Abstract

**Objectives:**

Existing research recognizes the critical role that social, sociodemographic, and acculturative processes play in increasing vulnerability for experiencing psychological distress among second-generation migrants. However, to date, far too little attention has been given to the study of psychological distress in this social group. The main objective of this study is to examine cultural identities, as well as expressions and causes of psychological distress among second-generation Albanian-speaking migrants in Switzerland.

**Methods:**

Semi-structured qualitative interviews were conducted with 13 Albanian-speaking participants between 19 and 35 years of age using the interview of the Barts Explanatory Model Inventory. Data were analyzed by qualitative content analysis using MAXQDA-2018 software.

**Findings:**

Study participants showed bicultural identities, which caused value conflicts and a feeling of being “caught” between Swiss and Albanian culture. Some participants experienced the fear of disappointing their parents. Others find it difficult to deal with conflicting norms and values. Parentification was another important cause within their cultural concept of distress. At the same time, (implicit) social support, i.e., spending time with the family and the community, was an important coping mechanism.

**Conclusion:**

Second-generation immigrants are exposed to specific risk factors for psychological distress. A better understanding of these risk factors and their coping mechanisms is essential for providing them with meaningful support services, both in prevention and psychotherapy.

## 1 Introduction

First- and second-generation migrants in Europe and elsewhere face specific challenges which may affect their mental health and well-being (e.g., Belhadj Kouider et al., 2014; Sarrasin et al., 2019). In a community survey with 1,239 participants in Switzerland (Steinhausen et al., 2009), young migrants showed significantly higher scores on internalizing and externalizing problems than natives. Especially youth with origins in South and South-East Europe showed more negative life events, more avoidant coping styles, more negative self-related cognitions, and less positive perceived parental behavior. In a more recent study that used data from the Swiss Household Panel (Sarrasin et al., 2019), young migrants aged around 20 reported lower well-being, more anxiety, sadness, and depressive symptoms than Swiss natives of the same age group.

There are various explanations for the higher risk of psychological distress and mental disorders among young migrants. The term “psychological distress” is used for non-specific symptoms that do not reach the level of a diagnosis (Patel et al., 2018) and, in cultural clinical psychology, as a term that is not linked to specific diagnostic categories (e.g., Nichter, 1981; Chentsova-Dutton and Ryder, 2020). Youth and young adulthood have shown to be a critical phase for the onset of mental disorders in general (Kim-Cohen et al., 2003). In addition to normative developmental tasks, second-generation migrants are often confronted with challenges such as discrimination (De Freitas et al., 2018), problems with family functioning and parents (Flink et al., 2012), identity development within two or more different cultures (Belhadj Kouider et al., 2014), and peer problems (McMahon et al., 2017). In Swiss and European studies (Belhadj Kouider et al., 2014; Sarrasin et al., 2019), the identified risk factors were living in a family with low economic status, parents’ uncertain cultural identity, and family function or parenting.

Among second-generation migrants, parentification can be another critical factor for psychological distress. Parentification describes the temporary or constant subjective assumption of the parental role by children. It was originally described in families with parent(s) with mental disorders (e.g., Aldridge and Becker, 2003), but has also been observed in migrant families (Hooper et al., 2011; Titzmann, 2012). Children, adolescents, or young adults take over parental roles due to the parents’ lack of socio-cultural competencies, such as language skills or knowledge about the education system (Titzmann, 2012).

In addition, the process of acculturation may foster or hinder mental health among migrants. Acculturation refers to the process of change and adaptation when individuals come into contact with other cultures (Gibson, 2001). Individuals may adapt to some aspects of a culture (e.g., general rules and behavior) while discarding others (e.g., values). Moreover, individuals may adapt to a new culture without necessarily neglecting their culture of origin. Biculturalism, i.e., adoption of receiving culture while retaining the culture of heritage, is associated with most favorable psychosocial outcomes, particularly among young migrants (Schwartz et al., 2010; Berry and Hou, 2017).

One important aspect of acculturation refers to the concept of the self. Research has brought forward the distinction between the “independent” vs. “interdependent self” (Markus and Kitayama, 1991). In independent cultural contexts, the main cultural tasks are autonomy, self-esteem, and uniqueness. People’s behavior is guided by their own attitudes, motivational goals, or values. In interdependent cultural contexts, the self is characterized by a strong commitment with the family and the community at large. The corresponding cultural concepts are relatedness and having harmonious relationships. Second-generation immigrants in Europe are potentially confronted with an independent cultural context outside their families, and interdependent cultural values within the realm of their family and community of origin.

The present qualitative study focuses on second-generation Albanian-speaking migrants in Switzerland. It aims to describe their cultural identity, perceived stressors, as well as symptoms of psychological distress, within their cultural and societal context. From a public health perspective, it is important to better understand their specific living conditions, stressors, and resources, with the aim of developing targeted interventions to enhance their resilience and promote their mental health and well-being.

According to the Swiss Federal Statistical Office (Bundesamt für Statistik, 2019), Kosovars are the fifth largest migrant group in Switzerland (approx. 107,000), and Albanian language is the third most common non-native language spoken in Switzerland after English and Portuguese, including about 222,000 people (Swiss Federal Statistical Office, 2019). Migration of Albanian-speaking individuals to Switzerland can be divided in three phases, i.e., labor migration (1960s), family reunification (1980s) and asylum-based migration (1990s) (Dahinden, 2005). This mixture between labor and asylum-based migration is special when compared to other immigrant groups.

A prior study examined cultural concepts of distress among first and second generation immigrants in Switzerland (Shala et al., 2020). Cultural concepts of distress include “idioms of distress” (Nichter, 1981), i.e., symptoms and different ways of expressing distress, and “explanatory models” (Bhui and Bhugra, 2002), i.e., assumptions about causes, consequences and treatment of psychological distress, in different cultural groups (Lewis-Fernández and Kirmayer, 2019). “Cultural identities” are part of such cultural concepts of distress (e.g., Rüdell et al., 2009).

In the previous study by Shala et al. (2020), large differences were observed between the first and second generation in terms of their understanding and expression of psychological distress, suggesting that these are not the same “cultural group.” This is in line with current definitions of “culture,” which include aspects such as age, gender, socio-economic or migration status in addition to ethnic origin (Markus and Hamedani, 2019). Therefore, we conducted additional interviews with the second generation to better understand their concepts of distress. Due to the different migration phases described above, this “second generation” includes a wide age range and different family histories regarding the migration process due to the different waves of immigration to Switzerland.

## 2 Study aims

This qualitative study explores cultural concepts of distress among the Albanian-speaking second-generation in Switzerland. Following the Barts Explanatory Model Inventory (BEMI, Rüdell et al., 2009), the present study explored the following two research questions:

- How do Albanian-speaking second-generation immigrants in Switzerland describe their cultural identity?
- How do they perceive and express psychological distress?
- What social and cultural factors foster psychological distress in this group?
- What strategies for coping and treatment do they prefer?

## 3 Materials and methods

### 3.1 Participants and recruitment

The study was approved by the ethics committee of the Canton of Zurich, Switzerland, in February 2017 (BASEC-Nr. 2016-02218). Participants were recruited through general practitioners, cultural associations, and social media (i.e., Instagram, Facebook). The study by Shala et al. (2020) showed that most of the younger participants (<40 years) preferred the German language in the interviews to talk about psychological distress. As language plays a fundamental role in exploring cultural concepts of distress, the age range for the present study was set at 18–40 years. This age range also largely corresponds to the age of the second generation. Recruitment material specified that we were looking for individuals with elevated levels of psychological distress. However, no formal screening took place to include the full spectrum of psychological distress.

### 3.2 Procedures and measures

A total of thirteen interviews were conducted, six in July and August 2017, and seven in July 2019. All interviews were conducted in German. Eleven interviews were conducted by the second author and two by the first author. Ten interviews took place at the Institute of Psychology in Zurich, two at the office of an Albanian-speaking general practitioner, and one interview at a participant’s home. Participants received no incentives.

The BEMI (Rüdell et al., 2009), German version (Shala et al., 2020), was used in this study. This semi-structured, qualitative interview includes 12 open-ended questions that cover five major topics: (i) cultural identity; (ii) perceived “symptoms,” complaints, and labels; (iii) cause/etiology; (iv) timeline, course of symptoms, and consequences; and (v) control, cure, treatment. The BEMI is a mixed-methods instrument including qualitative and quantitative aspects, but only the qualitative part was used in this study. Interviews were audiotaped and transcribed verbatim in MAXQDA-2018 (VERBI Software, 2017). Semantic-content transcription was performed according to the rule system (Dresing and Pehl, 2017). The interviews lasted between 29 and 106 min, with a mean of 61 min.

### 3.3 Data analysis

Qualitative content analysis (Kuckartz, 2012) was conducted using the MAXQDA-2018 software (VERBI Software, 2017; Rädiker and Kuckartz, 2019). The analysis encompassed seven steps: (i) initial text work; (ii) development of main thematic categories along the topics of the BEMI; (iii) first coding round with main categories according to “consensual coding” by Hopf and Schmidt (1993), with three interviews being coded by the first and second author independently to validate the coding system and to enhance reliability; (iv) compilation of all text passages coded with the same category; (v) inductive determination of subcategories and development of a coding guide; (vi) second coding process with differentiated categories and code summaries; and (vii) simple and complex analytics as well as visualization in form of tables and concept maps. Following Rädiker and Kuckartz (2019), concept maps allow to visualize categories, subcategories and their relationships. Concept maps therefore have two important characteristics: they make it possible to present findings and can be used as a tool for further content analysis.

For intercoder reliability testing, the kappa value according to Brennan and Prediger (1981) was calculated by the degree of correspondence between coders regarding categories assigned to text passages. Independently coded interviews were compared and discussed, kappa was calculated in MAXQDA-2018 (Rädiker and Kuckartz, 2019). According to Kuckartz (2012), kappa values of 0.6 to 0.8 are considered good, and 0.8 and above are considered very good. The discussion of disagreements in the data session resulted in an intercoder reliability of Kappa 0.9. This value testified to the high reliability of the category system, which could then subsequently be applied to the remaining interviews. The assumptions about the causes, symptoms and treatment were developed based on the previous study by Shala et al. (2020). Both coders (IP, MS) have an Albanian background, speak the Albanian language and belong to the second generation themselves. Through their personal experience and previous research, they were already familiar with the emerging topics in the interviews. In addition, two others authors (EH, NM) were involved in data analysis sessions and three (EH, NM, AM) in the writing process to ensure objectivity.

## 4 Findings

### 4.1 Sample

A total of *N* = 13 individuals participated in the study, eight of them (61.5%) were female. Eight participants reported their origins to be in Kosovo, two in North Macedonia, two in South Serbia (Preshevo), and one in Albania. Eight were born in Switzerland, two in Kosovo, and three participants lacked information on place of birth, as sociodemographic data were not systematically collected in the first round of interviews. Eight individuals had completed apprenticeship training, three held a Bachelor’s, one a Master’s, and two a doctoral degree. One individual did not provide information on the highest level of education. The youngest participant was 19 and the oldest 35 years old (*M =* 26.0 years, *SD* = 4.6). Written informed consent was obtained from all individual participants included in the study.

### 4.2 Cultural identity

Participants described their cultural identity as being shaped by Swiss and Albanian values. Albanian values were prominent in their families. At the same time, they went to school in Switzerland, made local friends and became familiar with the Swiss culture. Some described a feeling of having to choose between one of two identities.

“I would say that many have the problem to not be sure […] which identity they have. […] Many have the feeling that they need to choose if they are Swiss, Albanian, or Kosovar. And then there are some, they say, “No, I am Swiss, period.” I do not know the others [Albanians] so well, I do not know the language [Albanian], so therefore I am Swiss. And then there are others that say “Yes, I am a Kosovar., because we have a new state.” I would say that they did not understand the concept of identity, because a state does not define your identity.” (male, 22 years, student).

One participant (female, 35 years, PhD candidate) described this as a kind of “split.” The younger she had been, the easier it had been for her to split between the different contexts. According to her account, one half encompassed her first experiences with intimate relationships, and the other half consisted of school, university, and family, thus represented the “model student.” She expressed that it had always been her wish to bring the two halves together.

An important aspect of this dual identity was related to language. Two participants perceived that their parents had prevented them from integrating in Switzerland by speaking Albanian at home. In general, the participants said that they would speak Albanian with their parents and grandparents, but the preferred language among the siblings was German, as they found it easier to communicate with each other in this language, especially when expressing emotions. This language gap was perceived as further contributing to participants’ “dual identity.”

“I can speak the language, […] unfortunately I do not know the traditions that well anymore. Because we spoke a lot of German at home, especially with the siblings. Because it was easier for us. […] The respect for others or specifically for elder people, this is something I got from the Albanian side, that you need to show a lot of respect. Or that the guest is king.” (male, 29 years, self-employed).

### 4.3 Symptoms of distress

Table 1 shows the answer of each participant to the first question of the BEMI asking participants how they name their distress. Participants most frequently mentioned everyday terms such as “concerns about/fear of the future,” “sorrow,” “being overwhelmed,” “hopelessness,” “feeling lost,” “sadness” and “confusion.” Four interviewees used terms that contained etiological assumptions: “Intercultural stress,” “Swiss daily stress,” “pressure and stress,” “stress.” Participant 7 describes “intercultural stress” as following:

**Table 1:** Description of emotional distress/disease.

Participant	Description
1	Concerns about future
2	Feeling overwhelmed
3	Hopelessness
4	Being lost
5	Scared of future
6	Concerns
7	Intercultural stress/self-imposed responsibility
8	Sadness
9	Being confused
10	Daily Swiss stress
11	Pressure and Stress
12	(self-imposed) Pressure
13	Stress

“[…] I believe every human has something as a social role […] Me and my generation a bit more, because we see, we can have another social role too. It is not just the role of the son but also the mediator between two generations, two cultures […] (male, 22 years, student).

Participant 12 chose “pressure” as their symptom of distress and perceived it as follows:

“Pressure to progress, pressure that I tell myself that I am nowhere yet, pressure […] also in regards to my parents, even though they do not put pressure on me, […], I think that I am very privileged with them, so I tell myself, do something, make them proud so they do not have to pay your studies a hundred times.” (female, 23 years, student).

**Figure 1:**
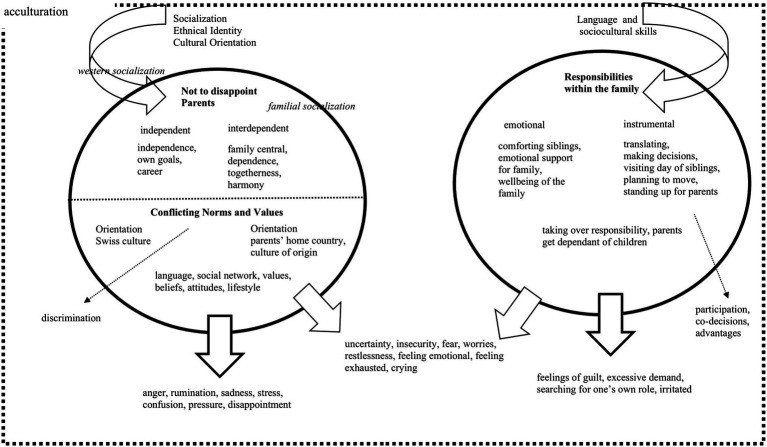
Concept map of the etiological factors and symptom descriptions and their relationship of Albanian-speaking young adults in Switzerland (19–35 years, *N* = 13).

Table 2 shows the symptoms that were mentioned by each participant. These symptoms were later grouped within the concept map (see Figure 1) according to the cause of distress that were named by the participants. The concept map thus gives an overview of the relation between causes and symptoms.

**Table 2:** Symptoms mentioned by each participant.

Symptoms	Participant												
	1	2	3	4	5	6	7	8	9	10	11	12	13
Anger	✔		✔					✔					
Uncertainty/Insecurity	✔				✔							✔	✔
Fear		✔	✔		✔						✔	✔	
Being overwhelmed		✔								✔			✔
Feelings of guilt		✔											
Rumination				✔					✔				
Sadness			✔		✔			✔				✔	
Hopelessness			✔										
Helplessness									✔			✔	
Psychological exhaustion/Indifference/Fatigue			✔							✔			
Barriers/Blockage/Burnout												✔	
Concern	✔				✔	✔						✔	✔
Shame												✔	
Heaviness												✔	
Stress											✔	✔	✔
Soul pain								✔					
Restlessness													✔
Being confused									✔			✔	
Depression	✔												
Aggression	✔												
Being emotional/agitated		✔								✔			
Being lost				✔									
Pressure											✔	✔	✔
Disappointment												✔	
Difficulties falling asleep/Sleep difficulties/Sleeplessness								✔			✔		
Gaining weight										✔			
Stomach Problems												✔	
Back pain	✔		✔										
Hadache													
Tenseness/Stiffness	✔			✔					✔				
Knee pain	✔							✔					
Not being able to move			✔										
Exhaustion/Fatigue					✔		✔		✔		✔	✔	
Teeth grinding										✔		✔	
Withdrawal/Rest	✔		✔			✔					✔		
Alienation					✔								
Searching for one’s own role					✔								
Wish to not be dependent from others			✔										
Farewell to a beloved person						✔							
Feel responsible											✔		
Hit the wall													
React touchy, more irritably, more extremely								✔	✔	✔			
Escaping from a situation	✔												
Crying	✔	✔					✔						

### 4.4 Causes of psychological distress

Participants indicated a variety of causes for their psychological distress. Thematic categories that concluded from their answers were: responsibilities within the family, not to disappoint family, and conflicting values and norms. Table 3 shows the category mentioned by each participant. As the table shows, two participants only mentioned one cause (participants 1 and 4) while the remaining 10 participants mentioned several causes. One participant (number 10) mentioned a cause for his distress that was not related to one of these three categories. The participant considered adult life in Switzerland as a main cause for his symptoms. All other participants mentioned causes for their distress that could be assigned to one or more of the aforementioned categories. The following subchapters specify each category and give examples of the conducted interviews.

**Table 3:** Causes for distress mentioned by participants.

	Causes		
	Responsibilities within the family	Not to disappoint parents	Conflicting norms and values
Participant			
1	✔		
2	✔	✔	✔
3	✔		
4			✔
5	✔	✔	✔
6	✔		
7	✔		✔
8	✔		✔
9	✔		
10			
11	✔	✔	
12	✔	✔	
13	✔	✔	

#### 4.4.1 Responsibilities within the family

A majority mentioned responsibilities in the family as their main source of distress. They described fulfilling administrative tasks in the family’s home, such as reading and translating letters, managing the household budget, getting involved in housing and moving, etc. In addition to such administrative tasks, some also assumed parental tasks for younger siblings. For example, one participant went to an interview with her sister’s supervisor to save her from losing her apprenticeship, while another participant went to a parents’ evening at the primary school of his younger brother. Participants were also involved in decision-making within the family or felt responsible for educating their parents regarding their rights and obligations.

Participants had taken over these tasks and responsibilities because of their language skills, the fact that they were familiar with the norms and values in Switzerland, and their knowledge of the education system when compared to their parents.

“[…] so I do not see it as something Albanian-specific, but I see it as…a typical guest worker family structure, that the children often take over things that the parents would normally take over, simply because they might have a better knowledge of the language, for example, but also because they have a better knowledge of socio-cultural [skills] or whatever.” (female, 35 years, PhD candidate).

One person explicitly stated that she was perceived as a third parent in the upbringing of the younger sister. Due to the conflicts between the parents and the younger sister, she was repeatedly asked by the parents to talk to the sister and persuade her of the parents’ views.

Some participants described perceiving or anticipating others’ emotions and therefore felt responsible for protecting the family harmony. As an example, one participant (female, 24 years, architect) feared that it would “destroy” her parents if her younger sister moved out. She also feared that the younger sister would distance herself from the family by doing so and asked her to communicate more with the family and to get closer to them. She expressed the need to prioritize her parents’ well-being over the sister’s or her own well-being, even though she understood the needs of her younger sister.

Several participants described that they took care of the psychological well-being of their mother. They would take time for the mother, talk to her, seek distraction, and even accompany her at work to relieve her of the burden. Due to a former illness of the mother, one person said that he would make sure that the mother would not exert herself, get enough rest and keep herself physically fit. Another person said he was there for the mother because the maternal grandparents were not well, and the mother was very worried. Maintaining emotional stability even went beyond the core family, as participants indicated that they were a point of contact for aunts and the rest of the family. One participant (female, 23 years, student) said that she would perceive herself as an “emotional hold” for family members. In the sample, it was noticed that mainly the oldest siblings took over such responsibilities. If not, it was often the child who still lived at home, independently of its order as a sibling.

According to participants’ reports, seeing the mental health of their parents or siblings at risk caused negative emotions and concern. They considered it to be the task of the close environment to take care of persons under stress, and they perceived their own well-being as closely intertwined with the well-being of family members.

“When they [family] are not doing well, that is what weighs on me emotionally. I know they are not doing good, and I know I cannot help them. Then I think it is going to be better soon. Personally, I become rather sad if I see that someone else is sad. And as soon as I see my mother getting better, I get better too.” (female, 22 years, childcare specialist).

This mutual interdependence went so far that individuals felt pressured to be always present, even if they themselves did not feel well. Furthermore, participants did not share their worries or discomfort with their parents, siblings, or close friends, due to a fear of being an (additional) burden to them.

“[…] more concerns about my environment, that I am not there for them just because I do not feel good. I have the feeling that even in situation where I am not doing good, I have to be there for others. “(female, 24 years, architect).

“[…] I did not tell her everything. For example, I did not tell her, that I had difficulties with sleeping or that, I did not want them add concerns to the ones they already had, that she does not have to concern about me too. […] I really find it difficult within the family, especially with the parents.” (female, 24 years, student).

In contrast, participants also reported positive effects of coping with their complaints by gathering with the family. In stressful situations, some wanted to go home to their parents for a few days to refuel their energy (see 4.6).

“[…] To go to my family. I do not live with them anymore because I’m studying. […] So, if I do not go home to my parents for a month or maybe longer it is, it gets another burden as well, because I then think, if I could only go home for two days, that would do me really good.” (female, 24 years, architect).

#### 4.4.2 Not to disappoint family

Several participants described the fear of disappointing their families. This was particularly evident in the form of a conflict between the lack of agreement between their own needs and the needs of the family or specific family members. This central role of the family appears in statements such as: “[…] my brother is one of the most important people in my life.” (female, 35 years, PhD candidate) or “[…] so as a community, as a family, this is more coming from, I think it is coming from the clan. That is the highest form of organization you have.” (male, 22 years, student). Another participant said: “My family is the most valuable thing that I have” (female, 23 years, student). The following statement shows the interpersonal conflict a participant was faced with, which emerged from conflicting needs between her and her family. The conflicting needs themselves became relevant, because family has a high importance for the participant:

“For me, it has always been clear, because without a family it does not work, so when it comes to the point, where I think I just want to [marry] this person, then I will fight until my parents buckle.” (female, 35 years, PhD candidate).

The influence of Albanian tradition on partnerships, marriages and family life seems to be another issue that concerned about half of the participants. Women in particular reported feeling pressured by her parents to marry an Albanian man. Three of the interviewed female participants (24, 30, and 35 years) kept their relationship to their Swiss or German boyfriends a secret, since they knew their parents would prefer an Albanian partner for their daughters. Consequently, some participants kept their relationship to themselves, one even said “to live a second life.” Not only parents’ views, but the opinion of grandparents and the extended family such as uncles and aunts is often considered as well. The family’s opinion on choice of partner put especially female participants under pressure, leading some of them to have a secret relationship with men from other nationalities. One participant feared telling her parents about this relationship, perceived to have no freedom and felt that any decision-making power lay with the parents.

“[…] Because I do stuff that my parents do not know of. And then I start thinking if this is really the right thing to do. Should I really move out, should I stay with my boyfriend or should I not better … date an Albanian guy … and then thoughts from earlier in life come up, such as insecurity. And I think the thoughts are not doing me any good. Because then it seems hopeless.” (female, 24 years, architect).

She sought therapeutic help and perceived that the therapist worked toward more independence from her parents, which was not in line with her own expectations:

“Well, for a long time it was about how do I get away from home […] somehow not focused on me at all, but more focused on how do I get away from home? As if this was the solution to get rid of my problems. And at some point, we found out that it does not work because I did not want to get away from home.” (female, 24, architect years).

In addition to nationality, one male participant (22 years, student) reported that for his parents it is also important to find someone of the same “social class.” According to another participant (female, 22 years, childcare specialist), parents would gossip when the offspring in other families married partners of other ethnicities. Parents would explain that marrying someone from another ethnicity, “that’s not us,” thus, not part of Albanian culture. This stands in sharp contrast with the wishes of three female participants (24, 30, and 35 years) who already had relationships with non-Albanian men and did not want a “typical Albanian wedding.”

Participants also expressed their need not to disappoint their parents with work- or study-related failures, even if their parents did not put pressure on them explicitly. The fact that their parents had migrated to Switzerland to offer their children a better future, often leaving higher education and social status behind to work under precarious conditions, implicitly urged participants to succeed in their own careers.

“I think there are several situations, on the one hand one always wants to be perceived as good by the parents, so to not disappoint them. Because you know that your parents came here to offer you a better future, you want to approach them by achieving something. My mother has always studied with us, [she] always wanted us to study or just do something, to follow our dreams. I guess mainly because I do not want to disappoint my parents personally and also because I have the feeling that they are so proud of my eldest sister (who achieved a master’s degree) so that we others have to achieve the same, so they get proud of us as well.” (female, 24 years, student).

Another female Participant of the same age expressed how the disappointment was expressed by her father and that feelings of guilt were induced through his behavior:

“[…] And I did not pass the trial period and he has been disappointed to the point where he did not speak to me for about three months. […] That was the first thing, that hit me really hard and it stuck with me until today, because with 12 years old, you do not expect something like this. […] I did not know how to deal with this. It really hurt me […] it made me feel guilty.” (female, 24 years, architect).

Two other respondents (female, 23 years; male, 27 years, both students) described having wished for a break after high school graduation. However, they felt pressured and immediately continued with their higher education, to make their parents feel proud. One participant (male, 27 years, student) used the metaphor “I have my parents on my neck” to describe this pressure.

#### 4.4.3 Conflicting values and norms

In the interviews, various text passages indicated that participants were partly caught in a conflict between the Swiss and Albanian cultural values. This was perceived more as an internal conflict because characteristics of the different nationalities were perceived as colliding. Due to the cultural differences, the individuals expressed a feeling of “sitting on the fence.”

“I would say, that many, especially the younger generation, have the problem to not be sure which identity one has. […] Many people have the feeling that they need to decide if they are Swiss or Albanian or Kosovar or… and then, there are some that say: “I am Swiss, this is it.” […] I do not know the people [from back home] that well, I cannot speak their language properly, therefore I am Swiss. And then there are others who say: “Yes, I am a Kosovar because we have a new country.” (male, 29 years, shift supervisor).

In the following excerpt, a participant describes the search for affiliation in a religious community. After marrying a Swiss citizen, she first had limited contact with her family. She and her partner decided to have an Islamic wedding ceremony in a mosque, although both described themselves as non-religious.

“Well, at some point you realize that you are not completely free of religiosity partly. Simply because […] you somehow want to be part of a community. And for me it was like […] when I knew that I was having a child, it was even more important for me… it was always important, but then it was even more important to be a part of the Albanian community with this child… my community of origin.” (female, 35 years, PhD candidate).

Another perceived burden within the sample was the feeling of “being different.” A participant described the difficulties that his father once had with him because they adapted behavioral patterns that were seen as Swiss in the father’s perspective:

“[…] It was more that he could not accept that we were Swiss now. […] That we tell our opinion. To everyone’s face. […] An Albanian would not do that, just exactly telling the truth, telling your true opinion. Maybe, I do not know, my lifestyle, my life. I am a pretty open person, I accept a lot of things an Albanian could not accept.” (male, 29 years, self-employed).

Female participants also felt that women who were educated or had a career had no place in society in their country of origin and their parents’ views were often described as conservative and patriarchal. At the same time, participants mentioned that they could understand their parents’ values, and they tried finding their own position.

“What is tradition, what is too patriarchal? […] I guess, that’s a question many keep asking themselves, how much tradition is still up to date? And what if you give up too much [of it]?” (male, 22 years, student).

In addition to value conflicts in their family environment, participants felt that they were perceived to be “different.” A participant expressed this feeling within the peer group with the same cultural background that she met through the student association:

“[…] Everything they [the student association] did was Albanian. They ate Albanian, danced Albanian, did events that only were related to Albania(n), but did not for example point out the political-migrant situation in Switzerland in general. I was looking for a self-worth that was not defined by not being worthy because I am not Albanian but rather because I am simply cool, because I can do things.” (female, 35 years, PhD candidate).

The same participant described the same feeling of being different also within the native peer group. Especially Albanian women were “perceived through a cultural lens” by natives and that there were subliminal prejudices in conversations:

“People immediately start to think, if you as an Albanian have a family conflict, it will always be perceived through a cultural lens. And only God knows what people imagine. The conflict was severe, and it dominated my life for ten years but it’s not what people expect. My father does not have a black beard and hits us against the wall.” (female, 35 years, PhD candidate).

Another participant (male, 29 years, self-employed) saw the possible reason for the refusal of a bar license for his pub in his name, what he perceived as discrimination. Another one was also limited in finding work: “People from working families have fewer connections. You get less help from the outside.” (male, 27 years, student).

### 4.5 Symptoms and causes

In a next step, we grouped causes and symptoms of psychological distress. Figure 1 shows the concept map, which explains the relationship between the cultural identity, the perceived causes and expressed symptoms in our particular sample.

Acculturation was set as a frame in the concept map since the causes for distress that were mentioned by our participants were all to some extent related to different aspects of the acculturation process. Individuals in our study are confronted with two different cultures, as expressed in their responses to the interview question concerning their cultural identity. The arrow on the top left represents the socialization process, which includes the development of their bicultural identity and their cultural orientation. This process leads to concerns about disappointing their parents, and to conflicting values, both which are represented in the left circle. The two factors are closely intertwined, which is symbolized by one circle with a dashed lined separating those two factors. The fear of disappointing parents often emerged when perceived demands were integrated in participants own self-concept, whether conflicting norms and values merged from different aspects of life. Concerns about disappointing parents and conflicting values were perceived as major causes of psychological distress. “Conflicting Values and Norms” and “Not to Disappoint Parents” led to symptoms such as anger, rumination, sadness, stress, confusion, pressure, and disappointment. The symptoms listed right under the left circle with a thicker arrow are specific for those two causes. The thinner arrow leading to the symptoms between the two circles links these causes to the unspecific symptoms within this sample. The specific term “discrimination” was only used by one participant. Therefore, the association from conflicting values to discrimination is represented as a dashed line, showing a possible association which has been found in previous studies (Bhugra et al., 2014; Özlü-Erkilic, 2015) and should be addressed in future research.

The right side of the concept map represents the process of parentification. They have better language and sociocultural skills than their parents (as they were socialized and educated in Switzerland), which lead them to taking over responsibilities within their families. The construct of “Parentificiation” can be theoretically divided into emotional und instrumental (Williams and Francis, 2010). Participants who mentioned responsibilities within their family as a cause for their distress reported specific symptoms right under the thick narrow on the right side, such as feelings of guilt, feeling overwhelmed, searching for one’s own role and feeling irritated. Again, the thinner arrow links this cause (i.e., responsibilities within the family) with the unspecific symptoms in the middle. The positive effect of instrumental parentification (i.e., participation, co-decisions) was only mentioned by one participant, which is represented as a dashed line, meaning there is less evidence within the sample for such an association. Several symptoms were named throughout both groups and could therefore not be assigned to a specific cause: Uncertainty, insecurity, fear, worries, restlessness, feeling emotional, exhausted and crying.

### 4.6 Timeline, course of symptoms and (anticipated) consequences

Participants reported that their symptoms had lasted from three months to seven years. Only two participants saw the origin of their distress in events that happened 10 and 17–18 years ago. All but one person described the phases of distress as being cyclical. That is, the distress recurred from situation to situation or was tied to a temporal interval. The duration of the distress was often related to life events such as having children of one’s own, starting, or continuing education, getting married, etc.

The reported main negative consequences of their distress included uncertainty, anger outbursts, difficulty in making decisions, hopelessness, and confusion. Among the feared consequences were: Ending up in a wheelchair due to back problems, losing passion, making a family member unhappy or harming them, becoming seriously ill, or not being able to overcome fear. When asked for potential positive consequences related to their distress, participants mentioned personal strength and growth, motivation, stronger family cohesion, and coming to terms with the problem.

### 4.7 Control, cure, and treatment

Seeking or giving social support represented the most common form of coping. The support was mostly provided by the family (i.e., parents, siblings, own children, cousins), friends or the romantic partner. Only a few expressed the desire to talk about their symptoms or their emotional state. It was more a matter of discussing practical solutions for specific problems or learning from the experiences of others. For the majority, it was about being with others and distracting themselves or talking about other things. Being around people who had similar experiences was perceived as particularly helpful.

In addition, self-management strategies (e.g., sports activity, confrontation, behavioral rules) and seeking professional help (i.e., doctors and counseling services) were frequently used. Participants indicated that they would seek professional help if the situation worsened and when talking to family and friends would no longer help.

A minority indicated endurance (Albanian: “durim”; “it must go on,” “nothing can be done,” “endure”) and acceptance (“you have to put up with it,” “disappearing is not the goal”) as possible coping mechanisms.

## 5 Discussion

The present study explored psychological distress among Albanian-speaking second-generation immigrations in Switzerland, with a main focus on their cultural concepts of distress. Participants in the present studies mainly presented a bicultural identity (Berry, 1980). They preferred to speak German with siblings, but Albanian with their parents and grandparents. They visited their parents’ country of origin regularly and mentioned being familiar with its traditions and culture.

Different studies show that a bicultural identity has a beneficial effect on mental health (Schwartz et al., 2010; Berry and Hou, 2017). By contrast, participants in this study expressed feeling caught between cultures or feeling different from others, which suggests that their bicultural identity has not evolved entirely successfully. They perceived conflicting values with the older generation as a cause of their psychological distress. From a theoretical perspective, these value conflicts are related to the self-concept. In interdependent cultural contexts, to which Albanian culture belongs, people give emphasis on the needs of the family and the community. By contrast, independent cultural contexts, as in Switzerland, pursue autonomy and one’s own goals (Markus and Kitayama, 1991). Participants in our study felt more oriented toward the (independent) Swiss culture, but at the same time still felt committed to Albanian (interdependent) values. Participants undergoing such value conflicts described feeling angry, sad, stressed, confused, disappointed, under pressure or to ruminate. This result confirms previous studies (Dennis et al., 2010), showing that conflicting values can be a significant predictor of psychological distress, leading to depressive symptoms and/or low self-esteem (Giguère et al., 2010).

Participants also described being confronted with stereotypes or perceiving discrimination by natives, which further contributed to conflicting cultural identities. From time to time, they made the experience of not being part of the dominating (Swiss) culture, e.g., in the labor market. Such experiences can cause distress, as other studies also show (e.g., Berry and Hou, 2017). Symptoms such as uncertainty, insecurity, fear, worries, restlessness, feeling emotional and exhausted, as well as crying were non-specific for any of the mentioned causes within our sample.

Parentification was another source of psychological distress in this sample. Participants often described having to take over a variety of responsibilities due to parents’ lack of German language skills and limited sociocultural abilities. In addition to this instrumental parentification, the sample also revealed emotional parentification (e.g., comforting siblings, emotional support for family, maintaining the wellbeing of the family). Participants either had to deal with one or both forms of parentification within the family. They described having feelings of guilt, being overwhelmed, searching for one’s role and being irritable. Descriptions of emotional distress among parentified offspring has been found in previous studies (Oznobishin and Kurman, 2009; Williams and Francis, 2010). In our sample, especially older siblings adopted a mediating role between parents and younger siblings, acting as a “third parent.” At the same time, one participant in the present study stated positive effects of being parentified, i.e., participating in discussions and having the chance to (co-)decide within the family. This result is in line with findings by Titzmann (2012), where a positive relationship between purely instrumental parentification and positive psychological outcome emerged. In parallel with other studies (Dow, 2011; Dow and Woolley, 2011; Shala et al., 2020), the most reported forms of coping were seeking social support from family members (parents, siblings, own children, cousins), friends, or partner, followed by self-managing strategies and to a lesser extend endurance and acceptance. Instrumental support (finding solutions for the causes of suffering) was more important than emotional support (finding solutions for the causes of suffering). To avoid being a burden to their family or friends, participants suppressed or kept their distress to themselves because they did not want to burden their family. Such implicit social support (i.e., just being together with family or friends without discussing the specific problem) was perceived as a helpful coping mechanism as well. As discussed in Shala et al. (2020) and Dow and Woolley (2011) this might be perceived as paradoxical, social support however is perceived differently within this group than expected from an etic view. Dow and Woolley (2011) note that Albanian migrants mainly socialize with other Albanians in the community. However, as soon as family problems arise (especially mental disorders), they are kept secret to preserve the image of the family.

Participants in this study mainly used “Western” terms to describe their distress (see 4.3), in contrast to participants of the first generation in a prior study (Shala et al., 2020). Another difference between generations emerged regarding coping. First-generation immigrants often used “durim” (patience) as a form of coping. They considered suffering to be normal, given by God or fate. Consequently, treatment was seen as unnecessary or inappropriate. This form of coping could not be found in the present study. Participants rarely made statements in a religious context, but saw the burden as part of (everyday) life and assumed that problems could be solved. At the same time, and in line with prior studies, findings of this study show that there is a reluctance to use professional help for different reasons, such as not feeling understood by mental health professionals (e.g., Giacco et al., 2014),

## 6 Limitations

This study has several limitations. About half of the respondents (7 of 13) had a high level of education (Matura, Bachelor, Master, Doctorate) and the sample is not representative of migrants in Switzerland in terms of educational level (Swiss Federal Statistical Office, 2019). In addition, sampling bias cannot be ruled out, as most participants who registered were recruited through social media (*n* = 9). It might be that participants, who were more familiar with psychological constructs due their own interest or because of being in therapy themselves or someone from the family, were more likely to participate.

Eleven interviews were conducted by a female and two interviews by a male interviewer. It remains unclear to what extent gender of the interviewer influenced our findings. This should be considered in future studies. Random assignment or intentionally controlling for gender matching between interviewers and participants could be informative.

Another limitation emerged from the nature of the BEMI: Asking for the symptoms of distress (question 2) and consequences (question 7) were often perceived as similar or even the same leading to repeating answers. This made it difficult to provide extra information in regards to better understand what the perceived consequences are within their concept of distress.

## 7 Conclusion

Our findings suggest an interplay of acculturative, social, and psychological processes in the emergence of psychological distress among Albanian-speaking second-generation migrants in Switzerland. Causes of distress are migration-specific but differ from the etiological factors for psychological distress in studies of first-generation migration. Bicultural identity and parentification emerged as important etiological factors for psychological distress in this specific sample. Findings of this study highlight the importance of understanding and involving the perspective of young migrant adults. As other studies have shown, this specific group faces specific challenges during a critical phase for the onset of mental distress.

A variety of implications arise for cultural adaptation of interventions. Second-generation migrants do not need adaptations in form of lingual translation, since concepts and terms they use to describe their distress are similar to western concepts of psychological distress. The findings suggest that the degree of bicultural identity, cultural self-concepts, as well as the degree and form of parentification are crucial for psychological distress. Considering those factors in psychotherapy could help adapting existing interventions for second-generation migrants, leading to a stronger identification. Second-generation migrants might need support in the process of integrating the culture of the host country and their parent’s cultural background to develop a bicultural identity. Professionals working with migrants, and particularly with the second generation, need to appreciate their views, desires, and needs by accepting different cultural heritage can help them to cope with psychological distress. The interdependent self-concept becomes evident in second-generation migrants and makes family a central feature. The direct or indirect involvement of the family may prove beneficial to the therapeutic process. This suggest that a systemic approach within therapy could be more appropriate to understand but also to support second-generation migrants with a similar background as the sample studied.

And finally, a reluctance to use professional help became apparent in this study, which parallels prior studies. Due to the large Albanian population in Switzerland and Europe, members of this community have access to a broad network of people with similar experiences, in addition to their own family. This network can also be used for advocacy: It would be desirable to increase the Albanian population’s awareness about potential benefits of psychotherapy for coping with psychological distress on the individual and the family level.

## Data availability statement

The datasets presented in this article are not readily available because we did not obtain consent for reutilization of the data. Requests related to the dataset should be directed to EH, eva.heim@unil.ch.

## Ethics statement

The studies involving humans were approved by Ethics Committee of the Canton of Zurich, Switzerland (BASEC-Nr. 2016-02218). The studies were conducted in accordance with the local legislation and institutional requirements. The participants provided their written informed consent to participate in this study.

## Author contributions

IP: Conceptualization, Data curation, Formal analysis, Investigation, Writing – original draft, Writing – review & editing, Visualization. MS: Conceptualization, Data curation, Formal analysis, Investigation, Methodology, Project administration, Supervision, Writing – review & editing. NM: Supervision, Writing – review & editing. AM: Supervision, Writing – review & editing. EH: Conceptualization, Funding acquisition, Supervision, Writing – review & editing.
